# Acoustic Detection Module Design of a Quartz-Enhanced Photoacoustic Sensor

**DOI:** 10.3390/s19051093

**Published:** 2019-03-04

**Authors:** Tingting Wei, Hongpeng Wu, Lei Dong, Frank K. Tittel

**Affiliations:** 1 State Key Laboratory of Quantum Optics and Quantum Optics Devices, Institute of Laser Spectroscopy, Shanxi University, Taiyuan 030006, China; waitingonce@163.com (T.W.); wuhp@sxu.edu.cn (H.W.); 2 Collaborative Innovation Center of Extreme Optics, Shanxi University, Taiyuan 030006, China; 3 Department of Electrical and Computer Engineering, Rice University, Houston, TX 77005, USA; fkt@rice.edu

**Keywords:** quartz-enhanced photoacoustic spectroscopy, acoustic detection module, quartz tuning fork

## Abstract

This review aims to discuss the latest advancements of an acoustic detection module (ADM) based on quartz-enhanced photoacoustic spectroscopy (QEPAS). Starting from guidelines for the design of an ADM, the ADM design philosophy is described. This is followed by a review of the earliest standard quartz tuning fork (QTF)-based ADM for laboratory applications. Subsequently, the design of industrial fiber-coupled and free-space ADMs based on a standard QTF for near-infrared and mid-infrared laser sources respectively are described. Furthermore, an overview of the latest development of a QEPAS ADM employing a custom QTF is reported. Numerous application examples of four QEPAS ADMs are described in order to demonstrate their reliability and robustness.

## 1. Introduction

**Figure 1:**
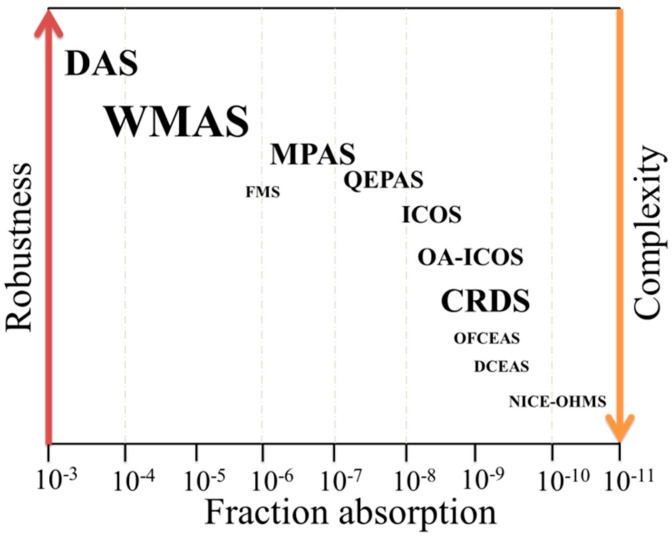
Different gas detection techniques are compared in terms of the sensitivity, complexity, and robustness of the sensor. The font sizes stand for their current importance for trace gases detection. DAS: direct absorption spectroscopy, WMAS: wavelength modulation absorption spectroscopy, MPAS: multi-pass absorption spectroscopy, FMS: frequency modulation spectroscopy, ICOS: integrated cavity output spectroscopy, OA-ICOS: off-integrated cavity output spectroscopy axis, CRDS: cavity ring-down spectroscopy, OFCEAS: optical-feedback cavity-enhanced absorption spectroscopy, DCEAS: direct cavity-enhanced absorption spectroscopy and NICE-OHMS: noise-immune cavity-enhanced optical heterodyne molecular spectroscopy.

The detection and quantification of trace gas has played an important role in the development and improvement of gas sensing technology. Gas sensing techniques find applications in many fields such as in agriculture, atmospheric science, environmental monitoring and medical diagnostics. Quartz enhanced photoacoustic spectroscopy (QEPAS), since it was first reported in 2002, has become one of the promising optical detection techniques due to the fact that it offers high detection sensitivity with a compact and cost-effective acoustic detection module (ADM) [1,2]. QEPAS is a variant of photoacoustic spectroscopy (PAS) [3,4], in which a quartz tuning fork (QTF) is employed instead of a microphone as a resonant acoustic transducer to detect photoacoustic signals. The replacement avoids the use of traditional gas-filled photoacoustic cells and thus removes restrictions imposed on the gas cell design by the acoustic resonance condition [5,6,7,8]. The QEPAS technique is compared with the other optical detection techniques in terms of sensitivity, complexity and robustness, as shown in Figure 1 [9]. The detection sensitivity of the QEPAS is worse than those of the optical cavity-based detection techniques, while the QEPAS is more robust and simpler. Compared to the conventional optical detection techniques, such as direct absorption spectroscopy (DAS) and wavelength modulation absorption spectroscopy (WMAS), the QEPAS technique has a better detection sensitivity.

A QTF is the key component in QEPAS based sensors, which can convert the mechanical vibrations induced by sound waves into current signals by means of the piezoelectric effect of quartz. The current signals, that are proportional to the target gas concentration, are converted into voltage signals and are then amplified using a transimpedance preamplifier. Commercially available standard QTFs, originally developed as frequency standards for watches and clocks, have a typical resonance frequency at 32,768 Hz and a high ***Q*** factor (usually a ***Q*** ≈ 100,000 when encapsulated in vacuum and a ***Q*** ≈ 10,000 at normal atmospheric pressure) [10,11]. When a QTF is placed in the acoustic near-field regions of optical sources, the presence of a gas enclosure is not necessary, since the gas enclosure serves only to separate the gas sample from the surroundings [5,6,7,8]. With the further development of the QEPAS technique, an acoustic micro-resonator (AmR) is mounted on a QTF to confine sound waves and to strongly enhance the sound wave intensity by coupling acoustic waves between the AmR and the QTF. The QTF and the AmR form a QEPAS spectrophone [8,12,13,14,15]. So far there are four different AmR configurations in terms of the structure of a QEPAS spectrophone: (i) an on-beam AmR configuration (two stainless steel tubes distribute symmetrically at the ends of the QTF along the axis perpendicular to the plane of the QTF.); (ii) an off-beam AmR configuration (a single tube with a small slit made in the middle is placed on the side of the QTF prongs in parallel, and this small slit faces to the QTF prong gap.); (iii) a T-shape AmR configuration (the T-shaped AmR consists of a long main tube and a short branch tube. The branch tube is perpendicularly intersected with the main tube in the middle of the main tube. The QTF is placed at the end of the branch tube to “off-beam” probe the photoacoustic signal excited inside the main tube) and (iv) a single-tube on-beam AmR configuration (a signal-tube AmR with two small slits in the middle was vertically placed between the prongs of a custom QTF.) [16,17,18,19,20,21,22,23]. In the four different configurations, the geometrical parameters of the AmR tubes must be optimized for the optimum detection sensitivity. The discussion in this paper relates only to the on-beam AmR configuration due to its high signal-to-noise ratio (SNR) resulting from the strong coupling between the AmR and the QTF.

An ADM consists of a spectrophone enclosed by a gas enclosure, which is equipped with a pair of windows as well as a gas inlet and outlet connector. The ADM is considered to be the core part in a QEPAS trace-gas sensor, due to the fact that its construction directly determines the performance of the QEPAS trace-gas sensor. This paper reviews several QEPAS ADMs designed for different QTFs and for different applications. The design considerations of an ADM are first discussed. Subsequently standard QTF-based ADMs for various applications including laboratory and industrial ones are described in detail. Finally, the latest ADMs using a custom designed QTF are reported.

## 2. Guidelines for the Design of an ADM

Several factors must be considered in the process of an ADM design. These factors mainly include the size of the ADM, the selection of a pair of optical windows, the means of supporting the spectrophone and the gas tightness of the ADM. The dimensions of an ADM mainly depend on the size of the spectrophone. Prior to 2013, a QEPAS ADM employed a commercially available 37-kHz standard QTF operating in fundamental frequency flexural mode. However, the beam from a light source with a wide beam diameter and a large divergence angle cannot clearly pass through the spectrophone, comprising an AmR and the 300-μm QTF prong spacing. Any blocked laser illumination results in an undesirable non-zero background due to the photo-thermal effect. This background may be several times larger than the thermal noise level of QEPAS and has a moving stripe-like interference pattern, which strongly impairs the detection sensitivity of the sensor [8]. Consequently, conventional QEPAS ADMs limit the use of some light sources such as light-emitting diodes (LEDs), vertical cavity surface emitting lasers (VCSELs), inter-band cascade lasers (ICL) and mid-infrared (MIR)/terahertz (THz) quantum cascade lasers (QCLs) [11]. In order to remove this restriction, custom QTFs with a large prong spacing were developed. Recently a custom QTF has been successfully applied to a QEPAS ADM with a terahertz QCL [24,25,26,27,28]. Since the resonant frequency of the custom QTF is different from the standard QTF, the optimal geometrical parameters of the AmR differ with respect to those of a standard QTF. 

**Figure 2:**
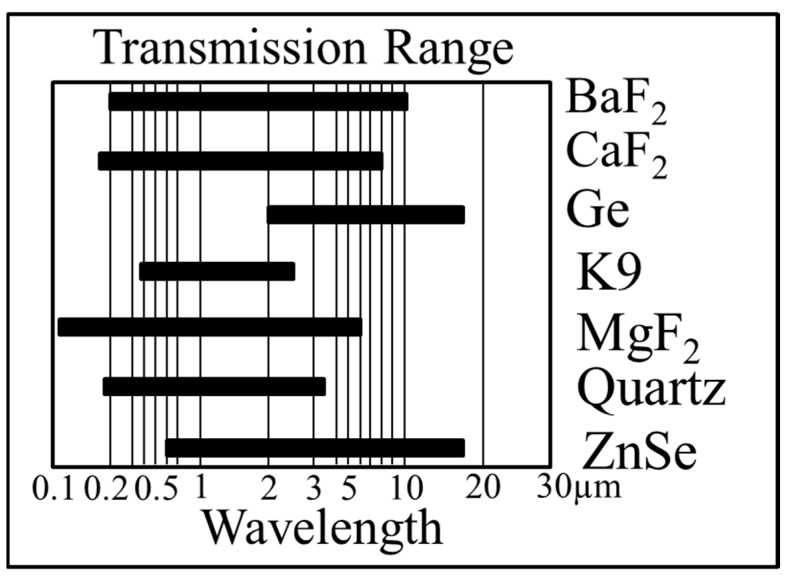
Comparison of transmission ranges for different window materials.

The optical windows of a QEPAS ADM require to have a high transmissivity in the wavelength range of the excitation light, which reduce a power loss and hence minimize window noise. The common window materials for a QEPAS ADM are quartz, calcium fluoride (CaF_2_), magnesium fluoride (MgF_2_), barium fluoride (BaF_2_), zinc selenide (ZnSe) and germanium (Ge), depending on the wavelengths of the used light sources. Figure 2 show the transmission wavelength ranges for different window materials. A quartz window is usually used in the ultraviolet wavelength region, while a K9 window is utilized in the visible wavelength region. For the wavelength region of 0.18–8 µm, a CaF_2_ window is appropriate. In the wavelength range of 5–16 µm, the window material must be changed to either ZnSe or Ge. In the THz region, high-density polyethylene or cyclic olefin copolymers are required.

**Figure 3:**
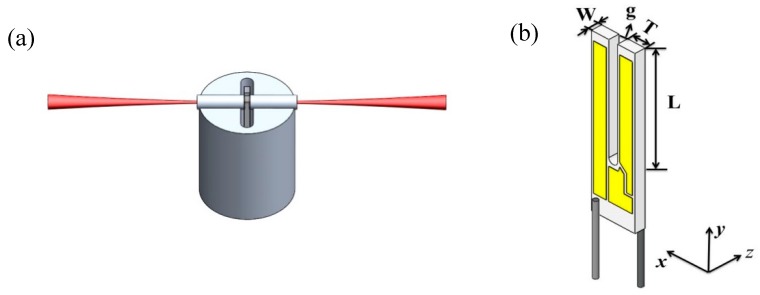
(**a**) A supporter to hold the AmR; (**b**) The length L, the thickness T, the width W and the prong spacing g of a standard QTF.

The relative position between the AmR tubes and the QTF has an important influence on the QEPAS SNR [29]. Therefore, the AmR tubes require an accurate assembly, which can be completed using a tray or supporter as shown in Figure 3a. Moreover, the gas tightness of the ADM is also crucial, since the different gases have the different relaxation rates, requiring different optimal gas pressures. Based on experimental studies, the optimal pressure for fast-relaxing molecules (such as water (H_2_O), propylene (C_3_H_6_) and sulfur hexafluoride (SF_6_)) is ~66.664 hPa [13,14,30]. which requires a good tightness for the ADM. Such a high gas tightness can be realized by means of a vacuum KF interface. With slow relaxation molecules, such as nitric oxide (NO), carbon dioxide (CO_2_) and carbon monoxide (CO), the optimal pressure is ~ atmospheric pressure [5,31,32,33]. A traditional O-ring seal is sufficient in order to implement gas tightness of the ADM.

The selection of excitation sources for a QEPAS ADM is also important, which can determine the detection sensitivity. A MIR laser source, such as a QCL or an ICL, can achieve a ppb-level detection sensitivity because many of the molecular species possess fundamental vibrational absorption bands in this spectral region. If employing a NIR laser source whose wavelength corresponds to overtone vibrational absorption band of molecular species, a ppm level detection sensitivity can usually be obtained. 

## 3. Standard QTF-Based ADM

### 3.1. On-Beam AmR Configuration of QEPAS Spectrophone

A commercial standard QTF is the most commonly used transducer in QEPAS, which has a prong length ***L*** of 3.0 mm, a prong thickness ***T*** of 0.4 mm, a prong width *W* of 0.33 mm and a standard prong spacing *g* of 300 µm, as shown in Figure 3b. In an on-beam configuration, a stainless-steel tube is cut into two pieces and the QTF is inserted between them, as shown in Figure 3a. The two AmR tubes should approach the plane of the QTF as much as possible, but not touch the QTF. An appropriate gap between the AmR and the QTF plane is 20~50 µm based on theoretical simulations and experimental verification [29]. The optimal geometrical parameters of the AmR for a near-infrared (NIR) beam are a single tube length of 4.4 mm and an inner diameter of 0.6 mm, achieving a SNR gain factor of 30 [29]. The center of the AmR is normally positioned at ~0.7 mm from the top of QTF [34]. However, with a MIR beam, the geometrical parameters of the AmR must be adjusted to the length of 3.9 mm and the inner diameter of 0.8 mm to allow the MIR beam to pass easily through the QTF prongs. The ADM should be designed to be large enough to accommodate a spectrophone. To date, three different standard QTF-based ADMs were designed based on the consideration of light beam quality, compactness, robustness and application scenarios.

### 3.2. Standard QTF-Based ADM for Lab Applications

**Figure 4:**
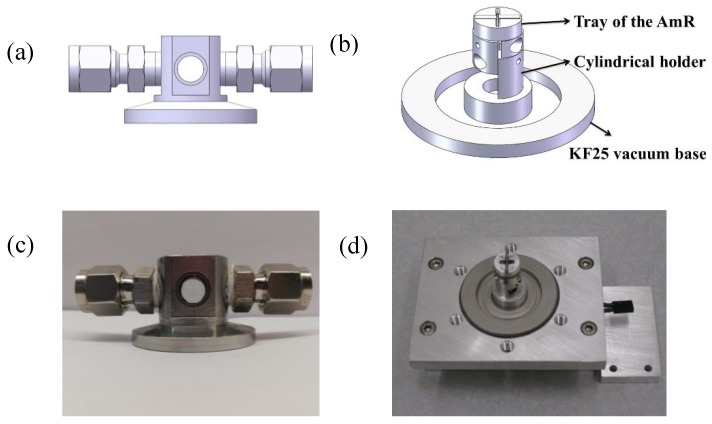
(**a**) CAD image of the KF25 vacuum cap; (**b**) CAD image of the KF25 vacuum base; (**c**) Photo of the KF25 cap; (**d**) Photo of the KF25 base.

A versatile ADM was developed for laboratory use to study the QEPAS technique. The ADM consisted of two KF25 vacuum components, a KF25 vacuum cap as shown in Figure 4a and a KF25 vacuum base as shown in Figure 4b. Both KF25 vacuum components were made of stainless steel with the advantages of low cost and good electromagnetic shielding. The cap was a KF25 vacuum flange which has a cylindrical space on the top with a diameter of 12.5 mm and a height of 20 mm. The cylindrical space was used to accommodate the spectrophone. A pair of 10 mm diameter windows was vertically glued to the front and back of the cap using Torr Seal. The incident and exit windows allowed the excitation light beam to pass through the ADM. When a KF25 vacuum cap is used, it should be rotated by an angle of 3–5° with respect to the incident beam in order to avoid the optical interference between two windows. The laser beam from the exit window was monitored by a power meter to verify that the laser beam passes completely through the spectrophone. A gas inlet and outlet were attached to both sides of the cap to enable the gas exchange inside the ADM.

The ADM base was a KF25 vacuum feedthrough flange, which allowed to send the electrical signal from the ADM. A QTF was clamped by two half cylinders and a holder with a height of 16 mm raised the QTF to the correct height. The two electrodes of the QTF were welded to two pins of the vacuum feedthrough flange. The AmR was placed in the grooves of a small cylindrical tray. The cylindrical tray with the AmR was adjusted to a pre-determined height, and then epoxy was used to fix the positions of the tray and the AmR. The KF vacuum interface can ensure excellent gas tightness with a pressure down to a few Pa. 

Figure 4c,d depict photos of the cap and the base. The developed ADM for laboratory use has the following advantages: (i) simple installation and disassembly, (ii) easy replacement of the QTF and AmR. The ADM for laboratory use has been successfully exploited for NIR and MIR sources. The standard QTF-based ADM was first reported in 2004 [35]. In this work a 1.53 µm fiber-coupled telecommunication distributed-feedback diode laser was used for a quantitative analysis of trace ammonia at 79.997 hPa. A detection limit of 0.65 ppmv and a normalized noise equivalent absorption coefficient (*NNEA*) of 7.2 × 10^−9^ cm^−1^W/$\sqrt{\mathrm{Hz}}$ was achieved. So far, the QEPAS sensor with this ADM has been employed to detect several gases in the visible spectral region (e.g., NO_2_) [36,37], in the NIR spectral range (e.g., NH_3_, CO_2_, CO, HCN, HCl, H_2_O, H_2_S, CH_4_, C_2_H_2_, C_2_H_4_) [5,30,32,33,38,39,40,41,42,43] and in the MIR spectral region (e.g., NO, N_2_O, CO, NH_3_, C_2_H_6_, (CH_3_)_2_CO and CH_2_O) [31,44,45,46,47,48]. Moreover, this ADM was also used in the development of the QEPAS technique, including the optimization of an on-beam QEPAS spectrophone [29], the application of an off-beam spectrophone [41], the realization of a double on-beam QEPAS spectrophone [49] and the position optimization of the AmR [34] and the invention of beat frequency QEPAS for fast and calibration-free continuous trace-gas monitoring [50].

### 3.3. Standard QTF-Based ADM for Industrial Applications

An ADM for industrial applications does not require easy installation and disassembly. Instead, the compactness and robustness are more important. Furthermore, a small ADM volume enables a faster gas exchange rate, thus resulting in a shorter response time. Two ADMs for industrial applications were designed for the NIR and MIR wavelength regions, respectively. 

**Figure 5:**
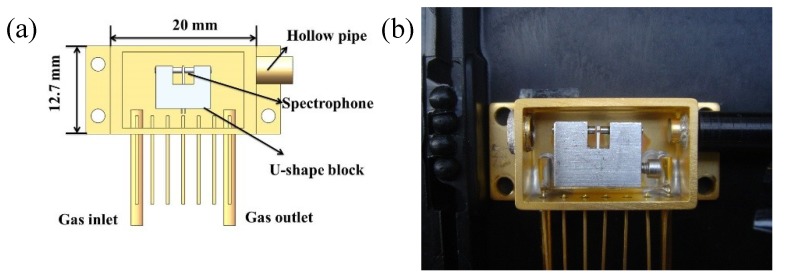
(**a**) CAD image of single-mode fiber-coupled ADM; (**b**) Photo of single-mode fiber-coupled ADM.

A single-mode fiber-coupled ADM was developed by Rice University (Houston, TX, USA) and Achray Photonics, Inc. (Ottawa, QC, Canada). Its CAD image and the names of all components are shown in Figure 5a. The spectrophone was enclosed by a telecom-style butterfly packaging, which has a comparable size with that of a telecom butterfly distributed feedback diode laser. The packaging was made from the nickel-iron alloy coated with a thin gold layer. The ADM has a rectangular shape with the dimensions of 20 × 12.7 × 8.5 mm^3^. A hollow tube was welded on the right-hand side of the rectangular shell to position the fiber focuser. On the left-hand side, a tilted angle optical window is equipped to allow the beam to exit the ADM. The tilted angle avoids light interference and reduces window noise. Two gas inlet and outlet tubes were attached to the downside of the rectangular shell, which allows gas flow in and out of the ADM. Between the gas inlet and outlet, there were a set of pins to output the QTF signal. Three clear holes were made on the edge of the shell base to fix the ADM. A U-shaped block, which acts as a spectrophone holder, was attached to the inner bottom surface of the rectangular shell. The position of the U-shaped block ensures that the focal point of the fiber focuser was positioned in the middle of the two QTF prong. There is a circular hole on the bottom of the U-shaped block to insert the QTF. The inserted QTF was clamped and positioned by a screw. A drill hole was made through the U-shaped block from the left side to the right side to place two 4.4-mm long hypodermic stainless-steel tubes with an inner diameter of 0.6 mm and an outer diameter of 0.9 mm. Figure 5b shows a photo of this fiber-coupled ADM.

Near-infrared laser radiation was transmitted to the ADM by means of a single-mode fiber focuser, in which a GRIN lens with a focal length of 10 mm was used. The laser beam exiting from the GRIN lens was focused through two 4.4-mm long hypodermic stainless-steel tubes and the QTF. The beam spot was ~100 μm between two QTF prongs. The ADM can operate at open or closed measurement modes. With an open measurement mode, a micro-pore hydrophobic PTFE filter membrane should be used on the top of the ADM in order to avoid contamination by dust and to filter out the water droplets. For such a measurement mode, a hermetic top lid is required to seal the ADM.

The ADM performance was compared with a state-of-the-art traditional photoacoustic cell by Dong [29]. A *NNEA* of 3.3 × 10^−9^ cm^−1^W/$\sqrt{\mathrm{Hz}}$ for C_2_H_2_ detection at atmospheric pressure was achieved. A QEPAS sensor using this type of ADM was first reported for simultaneous measurement of trace NH_4_, HCN, and C_2_H_2_ at 1.53 µm providing detection limits of ~100 ppbv at 599.981 hPa with a 4-s integration time [51]. Subsequently, this type of ADM was employed to the analysis of gas mixtures (H_2_S, CO_2_, and CH_4_), using two NIR, fiber-coupled diode lasers and resulting in a *NNEA* of 5.8 × 10^−9^ cm^−1^W/ and 5.3 × 10^−9^ cm^−1^W/$\sqrt{\mathrm{Hz}}$ for a dry and wet H_2_S, 1.1 × 10^−8^ cm^−1^W/$\sqrt{\mathrm{Hz}}$ and 4.0 × 10^−9^ cm^−1^W/$\sqrt{\mathrm{Hz}}$ for a dry and wet CO_2_, and 1.1 × 10^−8^ cm^−1^W/$\sqrt{\mathrm{Hz}}$ and 3.7 × 10^−9^ cm^−1^W/$\sqrt{\mathrm{Hz}}$ for a dry and wet CH_4_ [52]. Furthermore, a compact QEPAS multi-gas sensor based on this type of ADM was developed for the detection of CO, HCN, HCl and CO_2_ at atmospheric pressure, with detection limits of 7.74 ppm for CO, 450 ppb for HCN, 1.48 ppm for HCl, and 97 ppm for CO_2_ [53]. This type of ADM was also used to design a two-gas sensor for the detection of CH_4_ and NH_3_ in impure hydrogen (H_2_). The obtained *NNEA* values were 2.45 × 10^−8^ cm^−1^W/$\sqrt{\mathrm{Hz}}$ for CH_4_ at 266.658 hPa and 9.1 × 10^−9^ cm^−1^W/$\sqrt{\mathrm{Hz}}$ for NH_4_ at 66.664 hPa [54].

**Figure 6:**
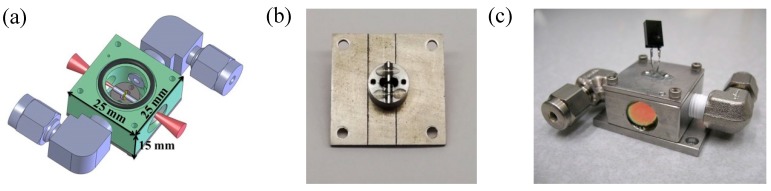
(**a**) CAD image of the free space MIR ADM; (**b**) photo of the top cap with spectrophone; (**c**) photo of the free space MIR ADM.

A fiber-coupled ADM is no longer feasible for a MIR QEPAS sensor due to the unavailability of MIR fibers, so that in this case a free space ADM must be considered. A concise cube structure was used as the shape of a free space ADM, resulting in ADM dimensions of 25 × 25 × 15 mm^3^, as shown in Figure 6a. The free space ADM cube was fabricated using stainless steel. A cylindrical chamber was made from the top to the bottom of the ADM cube in order to place the spectrophone. A gas inlet and outlet were installed on the right and left sides of the ADM cube. Two MIR windows were mounted on the front and back of the ADM cube with a tilted angle of 5°. Two O-rings were used on the top and bottom of the ADM cube to seal the inner chamber. The spectrophone was mounted on the top cap, as shown in Figure 6b. A small hollow cylinder as the AmR tray was fixed in the center of the top cap. Two grooves were made on the AmR tray to position the AmR tubes. A QTF passed through the AmR tray and exist from the tray surface. Two 3.9-mm long AmR tubes with 0.8-mm inner diameter were glued on the grooves of the AmR tray. The length and inner diameter of the AmR tube was shortened and enlarged, respectively, to match the MIR laser beam, so that the geometrical parameters of the AmR tube were no longer the optimal values. After the top cap was mounted on the ADM cube, the spectrophone was inserted into the chamber from the top of the ADM, as shown in Figure 6c. The output signal from the spectrophone can be obtained from the two pins of the QTF on the top of ADM. As a free space ADM, an active optical calibration was required to in order for the MIR beam to pass through the ADM.

The free space MIR ADM was successfully applied to detection experiments of CO, NO, and N_2_O. A NO sensor using a 5.26 µm MIR external cavity QCL was reported in 2011, with a detection sensitivity of 4.9 ppb with a 1-s integration time [55]. Based on this MIR ADM an ultra-sensitive detection of CO was achieved by using a 4.65 µm external-cavity QCL operating in continuous wave and pulsed modes, respectively. Experiments showed *NEC*s of 2 ppbv at a gas pressure of 133.329 hPa in the continuous wave mode and 46 ppbv at atmospheric pressure in the pulsed mode [56]. A sensor using this ADM with a 4.61 µm high power, continuous wave, distributed feedback QCL as the excitation source was demonstrated for the detection of CO and N_2_O. This experiment demonstrated a minimum detection limit of 1.5 ppbv at atmospheric pressure for humidified CO and 23 ppbv at 133.329 hPa for humidified N_2_O with a 1-s integration time [57].

## 4. Custom QTF-Based ADM

A commercially available QTF with a 32 kHz resonance frequency and 300 μm prong spacing has two restrictions for some specific applications, such as: (i) with slowly relaxing gases, the molecular relaxation cannot follow a high resonance frequency, leading to a weak signal of sound waves; (ii) a laser beam with a large beam waist is blocked by QTF prongs and produces undesired background noise. Therefore, QTF customization by varying the prong geometry is essential for QEPAS sensing applications depending on the specific laser source, target gas and sensing application. Some design efforts for the larger QTF prong spacing with a lower resonance frequency are in progress. The electro-elastic properties of all first generation of custom QTF were summarized and reported by Patimisco in 2016 [58]. The performance of several custom QTFs with different sizes was analyzed and reported by Sampaolo [59], Tittel [60], and Spagnolo [61]. There are six types of QTFs in the first-generation custom QTFs, whose parameters are listed in Table 1. Their advantages are that the larger prong spacing eliminates the collimation difficulty with a large diameter laser beam. A lower resonance frequency not only improves the detection sensitivity of slowly relaxing gases, but also allows the first overtones to be used due to the frequency reduction [62]. 

However, the challenge in QTF design is to reduce the resonance frequency while maintaining a high QTF quality factor and the low electrical resistance, since these QTF parameters significantly affect the QEPAS spectrophone performance. For the second-generation custom QTF, some modification techniques were introduced including shape, and prongs gap of custom QTF. For example, four grooves were added on the QTF surfaces; two QTF prongs were fabricated to have the shape of a hammer. The aim of those modifications was to improve the distribution of the stress field along the prongs by enhancing the weight near the top of the prongs, resulting in a decrease of the QTF electrical resistance while maintaining a low frequency and a high quality factor. The second-generation custom QTFs were reported by Patimisco in 2018 [63]. The parameters of the custom QTFs are listed in Table 2. These custom QTF were realized by starting from a Z-cut quartz crystal plate and a cutting angle of 2° with respect to the crystallographic X-axis, by using a standard photolithographic technique and chemical etching. Electrodes of opposite polarities were deposited on adjacent sides of the QTF prongs to collect the electrical charge.

**Figure 7:**
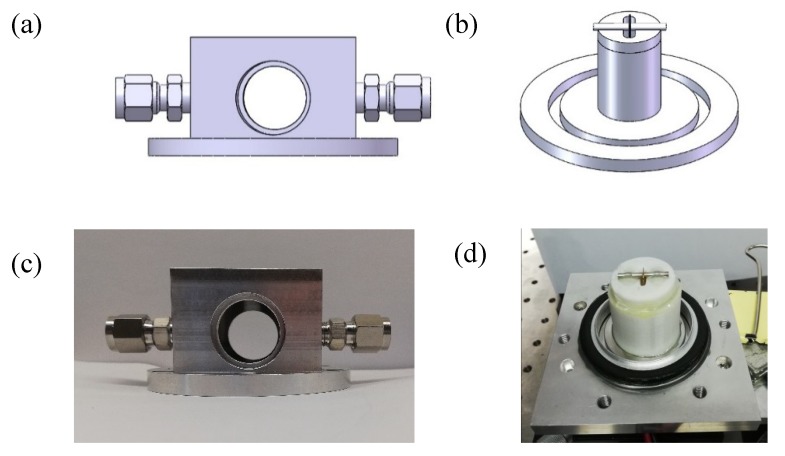
(**a**) CAD image of the KF40 vacuum cap; (**b**) CAD image of the KF40 vacuum base; (**c**) Photo of the KF40 vacuum cap; (**d**) Photo of the KF40 vacuum base.

A custom QTF-based ADM was designed following the structure of the standard QTF-based ADM for laboratory applications described in Section 3.2 in order to accommodate these large size custom QTFs and the AmR tubes. A vacuum cap was designed to provide a high vacuum, as shown in Figure 7a. The vacuum cap had a cuboid main body with dimensions of 57 × 57 × 35 mm^3^ and a KF40 vacuum interface. A cylinder space with a 42-mm diameter and a 38-mm height was provided inside the cuboid main body to accommodate the spectrophone. Two windows with a diameter of 25.6 mm were vertically glued to the front and back of the cap with a 5° angle with respect to two mounting surfaces, avoiding rotation of the cap during the assembly process. A gas inlet and outlet were equipped on the left- and right-hand sides of the cap to enable gas exchange inside the ADM. A KF40 vacuum base was employed as the counterpart of the KF40 cap as shown in Figure 7b. A hollow cylinder as a spectrophone holder was fabricated by means of 3D printing technology and glued onto the KF40 vacuum base in order to place and position the AmR tubes. Stainless steel was used for the custom QTF-based ADM. The photos of the KF40 vacuum cap and base are shown in Figure 7c,d, respectively.

The custom QTF-based ADM was applied to several experiments. A H_2_S gas sensor consisting of a custom QTF-based ADM and an optical fiber amplifying source was reported in 2015 [64]. The experiment, which used a QTF with an 800 µm prong spacing (QTF#2) and a 23 mm length of the AmR, shows a detection sensitivity of ~890 ppb with 1-s integration time. Due to the large prong spacing (QTF#2), a single-tube on-beam QEPAS technology was proposed based on the custom QTF-based ADM by H. Zheng in 2016 [21]. This method not only improves the detection sensitivity by two orders of magnitude, but also shortens the total length of the AmR, resulting in a significant size reduction in the spectrophone. After experimental optimization, the optimal length of the AmR was 26 mm. The NNEA, obtained from detected dry CO_2_ at atmospheric pressure, was 1.21 × 10^−8^ cm^−1^ ∙W/$\sqrt{\mathrm{Hz}}$. Using a custom QTF-based ADM, an overtone resonance enhanced single-tube on-beam quartz enhanced photoacoustic spectrophone was developed [22]. The AmR length was decreased to 14.5 mm due to a high overtone frequency of the custom QTF (QTF#5). This configuration produced an overall detection sensitivity enhancement factor of ~380 compared to the bare custom QTF. The custom QTF-based ADM was also used to demonstrate an experiment of double antinode excitation in 2017 [65]. Two identical AmR with a length of 9.5 mm were employed. As a result, a detection sensitivity gain factor of ~100 times was achieved, compared to the 1^st^ overtone resonances of a bare custom QTF. In 2017, a dual-gas QEPAS sensor system by means of the custom QTF-based ADM was realized and experimentally demonstrated [66]. Two beams from two independently modulated lasers were focused at two different positions between the QTF prongs to excite both the QTF fundamental and 1^st^ overtone flexural modes simultaneously. The proposed QEPAS methodology realized a continuous real-time dual-gas monitoring with a simple setup and small sensor size compared with previous multi-gas QEPAS sensors.

## 5. Conclusions

Since its first demonstration in 2002, QEPAS has proved to be a robust and sensitive trace-gas optical detection technique. The core component of a QEPAS sensor is an ADM which determines the performance of the QEPAS sensor. The size of an ADM depends on the dimension of the used spectrophone, which is determined by the used QTF. Therefore, the custom QTF-based ADM has a larger size than that of the standard QTF-based ADM. Moreover, the ADM design philosophy is different for different applications. A laboratory application requires simple assembly, easy disassembly and multiple functions, while industrial applications require reliability and robustness with few moving parts. Based on these design philosophies, we reported two ADMs using a standard and a custom QTF, respectively, for laboratory use and two industrial ADMs, one fiber coupled for a NIR excitation beam and free space for a MIR excitation beam. The four QEPAS ADMs meet the demands of a variety of experiments and can match the different laser sources. So far, they have been widely employed in numerous QEPAS experiments and for different application scenarios. A summary of the four QEPAS ADMs is depicted in Table 3 for fifteen trace-gas species which were detected using these ADMs. The results in the gray section, purple section, green section and red section are, respectively, from standard QTF-based ADM for laboratory applications, standard QTF-based and single-mode fiber-coupled NIR ADM for industrial applications, standard QTF-based and free space MIR ADM for industrial applications and custom QTF-based ADM.

## Figures and Tables

**Table 1:** Parameters of the first generation of the custom QTFs: Prong spacing (*g*), prong length (*L*), prong thickness (*T*), frequency (*f*) and quality factor (*Q*) in atmospheric pressure.

	*g* (mm)	*L* (mm)	*T* (mm)	*f* (Hz)	*Q*
QTF#1	0.4	3.5	0.2	14,049.2	7323.69
QTF#2	0.8	10	0.9	7230.27	18,654.18
QTF#3	0.5	10	1	8439.51	25,484.95
QTF#4	0.6	11	0.5	3456.69	8388.12
QTF#5	0.7	17	1	2869.07	11,901.88
QTF#6	1	19	1.4	4250.01	37,712.74

**Table 2:** Parameters of the second generation of the custom QTFs: Prong spacing (*g*), prong length (*L*), prong thickness (*T*), frequency (*f*) and quality factor (*Q*) in atmospheric pressure.

	*g* (mm)	*L* (mm)	*T* (mm)	*f* (Hz)	*Q*
QTF-S08	0.8	9.4	2	15,841.92	15,710
QTF-S15	1.5	9.4	2	15,801.66	15,400
QTF-S08-T	0.8	9.4	2	12,460.55	15,540
QTF-S08-G	0.8	9.4	2	15,222.93	15,050

**Table 3:** Four different ADMs-based QEPAS performance for 15 trace gas species. NNEA: normalized noise equivalent absorption coefficient, NEC: normalized noise equivalent concentration.

Molecule (host)	Wavenumber (cm^−1^)	Pressure (hPa)	Power (mW)	*NNEA* (cm^−1^W/Hz^1/2^)	*NEC* *(ppmv)*	Reference
CO (N_2_ + H_2_O)	4291.5	Atmospheric pressure	8.8	1.8 × 10^−5^	11.2	[5,30,44,45]
NO (N_2_)	1900.075	333.323	100	3.6 × 10^−9^	0.015	[31]
CO_2_ (air)	6361.25	Atmospheric pressure	40	2.2 × 10^−9^	29	[32,33,38]
NH_3_ (N_2_)	6322.45	Atmospheric pressure	1250	1.395 × 10^−9^	0.5	[35,38,39,46]
NO_2_ (N_2_)	2415.40	Atmospheric pressure	7	4.2 × 10^−9^	0.0013	[36,37]
H_2_O (N_2_)	7306.75	79.997	9.5	1.9 × 10^−9^	0.09	[38]
C_2_H_2_ (N_2_)	6523.88	Atmospheric pressure	57	4.1 × 10^−9^	0.03	[38]
H_2_S (N_2_)	6328.88	Atmospheric pressure	1250	1.525 × 10^−9^	1.6	[39]
HCN (air)	6539.11	79.997	50	4.6 × 10^−9^	0.155	[40]
H_2_S (N_2_)	6320.6	Atmospheric pressure	1.4	9.8 × 10^−9^	0.142	[41]
C_2_H_4_ (N_2_)	6177.14	Atmospheric pressure	15	5.4 × 10^−9^	1.7	[43]
N_2_O (N_2_ + 5%SF_6_)	2195.633	66.664	19	1.5 × 10^−8^	0.007	[44,45]
CH_2_O (N_2_)	2832.50	Atmospheric pressure	3.4	2.2 × 10^−8^	0.6	[48]
CO_2_	6493.42	Atmospheric pressure	21.1	16 × 10^−9^	0.692	[29,52,53]
C_2_H_2_ (N_2_)	6523.87	599.981	58.0	1.9 × 10^−9^	0.03	[29,51]
HCN (N_2_)	6539.11	Atmospheric pressure	35.5	5.3 × 10^−9^	0.45	[51,53]
NH_3_ (N_2_)	6528.80	599.981	62.0	6.9 × 10^−9^	0.1	[51,54]
H_2_S (N_2_)	6320.6	Atmospheric pressure	38.3	5.8 × 10^−9^	10.1	[52]
CH_4_ (N_2_)	6057.09	Atmospheric pressure	16.0	1.1 × 10^−8^	1.5	[52,54]
CO (N_2_ + 1.1%H_2_O)	4288.29	Atmospheric pressure	2.0	1.41 x10^−8^	7.74	[53]
HCL (N_2_)	5739.26	Atmospheric pressure	14.7	5.17 × 10^−8^	1.48	[53]
NO (N_2_ + 2.5%H_2_O)	1900.08	279.991	66	5.6 × 10^−9^	0.0049	[55]
CO (N_2_ + 2.2%H_2_O)	2176.28	133.329	71	1..48 × 10^−8^	0.002	[56,57]
N_2_O (N_2_ + 2.6%H_2_O)	2169.60	133.329	400	2.91 × 10 ^−9^	0.023	[57]
CO_2_ (N_2_)	6361.25	Atmospheric pressure	40	1.21 × 10^−8^	90	[21]
H_2_O (N_2_)	7306.75	Atmospheric pressure	13	1.73 × 10^−9^	0.23	[22,65]
H_2_S (N_2_)	6320.60	Atmospheric pressure	1520	1.29 × 10^−8^	0.89	[64]
